# Addendum: Uberti, F.; et al. Study of Magnesium Formulations on Intestinal Cells to Influence Myometrium Cell Relaxation. *Nutrients* 2020, *12*, 573

**DOI:** 10.3390/nu12072119

**Published:** 2020-07-17

**Authors:** Francesca Uberti, Vera Morsanuto, Sara Ruga, Rebecca Galla, Mahitab Farghali, Felice Notte, Chiarella Bozzo, Corrado Magnani, Antonio Nardone, Claudio Molinari

**Affiliations:** 1 Laboratory of Physiology, Department of Translational Medicine, University of Piemonte Orientale, via Solaroli 17, 28100 Novara, Italy; vera.morsanuto@uniupo.it (V.M.); sara.ruga@uniupo.it (S.R.); rebecca.galla@uniupo.it (R.G.); mahitab.farghali@uniupo.it (M.F.); felice.notte@uniupo.it (F.N.); claudio.molinari@uniupo.it (C.M.); 2 Laboratory of Applied Biology, Department of Translational Medicine, University of Piemonte Orientale, via Solaroli 17, 28100 Novara, Italy; chiarella.bozzo@uniupo.it; 3 Medical Statistics and Cancer Epidemiology Unit, Department of Translational Medicine, University of Piemonte Orientale, 28100 Novara, Italy; corrado.magnani@uniupo.it; 4 Department of Translational Medicine, University of Piemonte Orientale, 28100 Novara, Italy; antonio.nardone@uniupo.it

The authors would like to publish this addendum to their recent publication [1] to declare that Francesca Uberti and Claudio Molinari are co-founders of an academic spin-off of the University of Eastern Piedmont called noiVita s.r.l.s. None of the authors have obtained any commercial profit from this paper [1]. The Conflicts of Interest section of the backmatter should be written:

**Conflicts of Interest:** F.U. and C.M. are co-founders of an academic spin-off of the University of Eastern Piedmont called noiVita s.r.l.s. None of the authors have obtained any commercial profit from this paper. The rest of the authors declare no conflicts of interest.

We apologize for any inconvenience to the readers this oversight may have caused.
